# Measuring Women’s Empowerment in Agriculture: Innovations and evidence

**DOI:** 10.1016/j.gfs.2023.100707

**Published:** 2023-09

**Authors:** Agnes Quisumbing, Steven Cole, Marlène Elias, Simone Faas, Alessandra Galiè, Hazel Malapit, Ruth Meinzen-Dick, Emily Myers, Greg Seymour, Jennifer Twyman

**Affiliations:** a International Food Policy Research Institute, Washington DC, USA; b International Institute of Tropical Agriculture, Dar es Salaam, Tanzania; c Alliance of Bioversity International and the International Center for Tropical Agriculture (CIAT), Rome, Italy; d International Livestock Research Institute, Nairobi, Kenya; e Independent Consultant, Cali, Colombia

**Keywords:** Women's empowerment in agriculture, Gender equality, Food systems, Measurement

## Abstract

This paper addresses women's empowerment in agriculture, innovations in its measurement, and emerging evidence. We discuss the evolution of the conceptualization and measurement of women's empowerment and gender equality since 2010. Using a gender and food systems framework and a standardized measure of women's empowerment, the Women's Empowerment in Agriculture Index (WEAI), we review the evidence on “what works” to empower women based on impact evaluations of a portfolio of 11 agricultural development projects with empowerment objectives and a scoping review of livestock interventions. We then review the evidence on associations between empowering women and societal benefits--agricultural productivity, incomes, and food security and nutrition. We conclude with recommendations for measurement and policy.

## Highlights

• Women's empowerment and gender equality metrics needed to track progress towards SDG5.
• Conceptualization of empowerment matters for measurement.
• Agency is a focus in new multidimensional measures of empowerment.
• Quantitative and qualitative methods both important to understand empowerment impacts.
• Women's empowerment has both intrinsic value and positive social benefits.

## 1 Introduction

Women's empowerment and gender equality are central to gender-transformative change and a more holistic and inclusive way of approaching gender in agriculture. Previously viewed only as instrumental in achieving objectives related to health and nutrition (Sraboni et al., 2014; Galiè et al., 2019; Heckert et al., 2019), productivity (Diiro et al., 2018) and resource management (e.g., Sodhi et al., 2010), women's empowerment and gender equality are now regarded as goals of agriculture and food systems interventions in themselves (Elias et al., 2021).

Compared to women's empowerment, gender equality is relatively straightforward to conceptualize, and the increase in sex-disaggregated and intrahousehold data has expanded available data on gender equality in many areas. Empowerment is a more complex concept, and the choice of conceptual definitions of empowerment has implications for measurement. While different perspectives on the measurement of women's empowerment exist, the field has generally coalesced around a conceptual definition of empowerment based on the work of Naila Kabeer. Analyzing more than 9,000 peer-reviewed articles published on women's empowerment between 1999 and 2019, Priya et al. (2021) found that Kabeer's (1999) article in *Development and Change*, in which she defines empowerment as the process by which people expand their ability to make strategic life choices, particularly in contexts in which this ability had been denied to them, was cited more than any other article. Other definitions of empowerment exist, such as the typology of power (Rowlands 1995, 1997), which juxtaposes the notion of dominating or exerting “power over” others with generative forms of empowerment, including “power within” (involving self-respect, self-efficacy, and an awareness of rights), “power to” (enacting personal goals) and “power with” (acting collectively toward shared interests).

New thinking on agri-food systems has also led to recognition of the multiple relationships between women's empowerment and gender equality and food systems outcomes (Njuki et al., 2022). Although empowerment is the right of all individuals regardless of livelihood or location, we focus on women's empowerment in agriculture and food systems given the importance of women in these sectors. According to FAO's (2023) *The Status of Women in Agrifood Systems*, although working women's share of agricultural employment declined between 2005 and 2019 from 44% to 36% and men's from 47% to 38%, the decline is partly offset by an increase in non-agriculture food system employment. Women's share increased from 56% to 64%, and men's from 53% to 62%, with substantial variation across regions and countries (FAO 2023). Elevation of gender equality and the empowerment of women and girls to Sustainable Development Goal (SDG) 5 has also created the need for indicators to monitor progress. Assessing empowerment in agriculture and food systems contributes to women's empowerment and gender equality in several ways. Quantitative and qualitative assessments of empowerment can support holistic design of projects, programs, and policies. Assessments can also monitor whether and how initiatives such as projects, programs, policies or social movements and efforts led by women's organizations are contributing—positively or negatively—to women's empowerment. Measuring and/or assessing empowerment serves to build upward and downward accountability and credibility (Batliwala and Pittman 2010), and the assessment process itself can challenge power relations (Hillenbrand et al., 2015).

In this paper we describe how thinking about women's empowerment and gender equality has advanced since 2010. Based on a review of interventions, we examine what has been effective in empowering women. We review studies on the association between women's empowerment and such societal benefits as agricultural productivity, incomes, food security, nutrition, and environmental benefits. Finally, we make recommendations for measurement and policy.

## 2 Background

The Beijing Declaration (UN 1995) that emerged from the 1995 Fourth World Conference on Women included a focus on the advancement and empowerment of women, highlighting the need to challenge patriarchal and intersecting structures that subordinate women in society and create gender inequalities. While gender inequalities related to rights, resources and responsibilities in the agricultural sector have been well documented (see (FAO, 2011) and other articles in this special issue), less well documented are gaps in empowerment and agency between men and women, owing to the lack of individual-level data and the lack of consensus on how to define and measure these concepts. In particular, a lack of conceptual clarity around the term “empowerment” as mobilized in the international development agenda, along with the subversion of the term in neoliberal political agendas, has diluted the concept that social activists brought to the table in Beijing (Batliwala 2007; Cornwall and Rivas 2015; Nazneen et al., 2019).

Kabeer's conceptualization of empowerment encompasses three main elements: resources, agency, and achievements. On one hand, there is more evidence on gender equality in resources and achievements than on agency because of the existence of established metrics and rapidly increasing availability of sex-disaggregated, individual-level data. The typical achievements measured include poverty, income, wealth, nutrition/health (women's and children's), education, among others. While these measures of achievement provide information about gender gaps, they are not directly aligned with Kabeer's concept of empowerment, which is about goals that are unique to individuals. Measured achievements may be linked or associated with individual goals but may not provide a full picture of whether the person is achieving their own personal goals.

On the other hand, data on agency remains scarce, especially at the national level. Much of the existing data either comes from individual projects and/or is only representative at subnational levels. Agency is also arguably more difficult to measure. The most common way of measuring agency has been to consider women's (and men's) participation in different decisions, typically within the household. While this captures part of agency, it does not fully depict the concept of agency, defined as “the ability to define one's goals and act upon them” (Kabeer 1999, 438). Kabeer (1999) explains that while decision-making is often used to measure agency, it can also take other forms that are unobservable (and thus difficult to measure), such as negotiation, manipulation, subversion, and resistance, and is closely related to the idea of “power within” (Rowlands 1997).

Even the current use of participation in decision-making to measure agency has its drawbacks. Typically, women report their own participation (or ability to participate) in household decision-making processes. It is also often framed in terms of autonomous decisions that women make alone versus joint decisions made with spouses and/or others, with the highest level of women's empowerment often thought to be sole decisionmaking, and the lowest when she is not involved in decisionmaking at all (Bernard et al., 2020; Peterman et al., 2021; Seymour and Peterman 2018). However, whether this ranking is sensible is unclear; what reported joint decision-making means in terms of agency is ambiguous. In some cases, a joint decision could mean that women are gaining agency/voice/decision-making power—they are making decisions and acting upon their goals. In other cases, a joint decision may mean that someone else (a spouse, for example) has a say and could thus impede a woman's ability to make strategic choices. In Uganda, Acosta et al. (2019) found that joint decision-making can range from being informed (either before or after a decision has been taken) to participating in conversations about the decision.

Furthermore, decisions included in measures of women's empowerment are not necessarily related to women's own goals. Kabeer's definition of empowerment focuses on gaining the ability to make strategic life choices. This implies two things: first, a change over time— a transformation from not being able to make one's own choices to having that ability—and second, a focus is on strategic life choices, which implies focusing on one's own goals.

## 3 Emerging thinking around women's empowerment and food systems

Over the past 10 years, approaches and tools to measure gender (in)equality and women's empowerment in agriculture and food systems have proliferated. While efforts to assess empowerment were previously focused on qualitative understandings of empowerment, primarily from the perspectives of those whose empowerment was being assessed (emic perspectives), more recent efforts have attempted to quantify women's empowerment and shifts therein related to women's participation in agriculture, nodes of the agricultural value chain beyond production, and food systems more generally.

The complex, multidimensional, and context-specific nature of empowerment complicates its assessment. Increases in the availability of sex-disaggregated and intrahousehold data have improved assessment of the extent of gender equality in resources and achievements, but measuring agency is more difficult. Some measurement approaches capture changes in empowerment as a process, and others as an outcome (Carr 2003; van Eerdewijk et al., 2017). Assessing empowerment as a process is especially challenging because measurement is often attempted at one point in time but must capture forward and backward movements and trajectories.

Assessments should capture “different dimensions and sites of empowerment in a more holistic way, one that aims to understand the relational dynamics of power and positive change at a variety of levels, in different spaces and over time” (Cornwall 2016, 345). Most attempts to measure empowerment have collected cross-sectional data or asked respondents to recall their experiences retrospectively. However, panel data on empowerment outcomes are better equipped to examine longitudinal trajectories of women's empowerment and can complement qualitative assessments that focus on trajectories. Because internationally validated measures of women's empowerment have only recently been developed, there are only a few countries that have panel data on women's empowerment, but the Demographic and Health Surveys, for which there are multi-year observations, are often used to create panel data on proxies of women's empowerment, such as decisionmaking. Furthermore, the desire to measure across countries must be balanced with attempts to assess the contextual nature of empowerment (Richardson 2018).

**Fig. 1:**
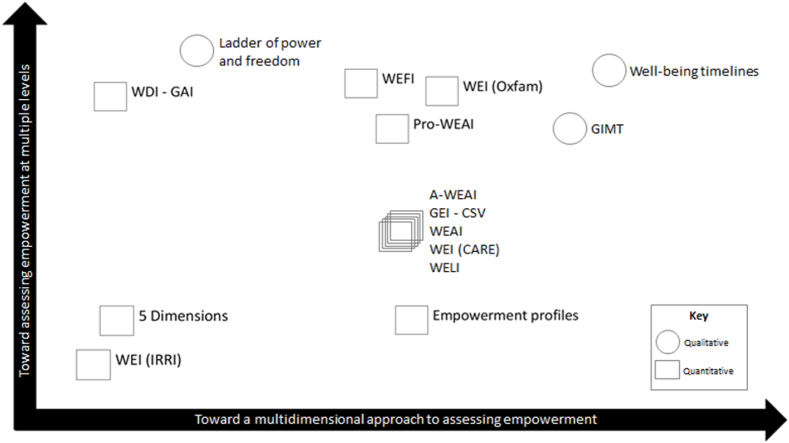
Mapping of empowerment measurement tools by dimension and level of empowerment Key: WEI (IRRI): Women's Empowerment Index, International Rice Research Institute; 5 Dimensions: Comparison of the Five Dimensions of Men's and Women's Empowerment; WDI-GAI: Women's Decision-Making Index and Gender Attitudes Index; WELI: Women's Empowerment in Livestock Index; WEI (CARE): Women's Empowerment Index (CARE); WEAI: Women's Empowerment in Agriculture Index; GEI-CSV: Gender Empowerment Index for Climate Smart Villages; A-WEAI: Abbreviated WEAI; Pro-WEAI: Project-level WEAI; WEI (Oxfam): Women's Empowerment Index (Oxfam); WEFI: Women's Empowerment in Fisheries Index; GIMT: Gender Indicator Monitoring Tool (CARE).

Source: Elias et al. (2021).

Tools for measuring empowerment can be clustered roughly into four groups: those that (1) focus only on one dimension (resources, agency or achievements) and assess empowerment at one level (individual, relationship or environmental); (2) focus on one empowerment dimension but at multiple levels; (3) use a multidimensional approach to assessing empowerment at one or more levels; and (4) explore the three dimensions of empowerment at the three levels of inquiry—personal, relational and environmental. A comprehensive review of these tools (Elias et al., 2021) provides insights into the current state of efforts to measure women's empowerment (Fig. 1).

We find that most of the reviewed tools recognize the multidimensional and multilevel nature of empowerment in assessments, which bodes well for acknowledging the complexity of the concept in agricultural research for development (AR4D) thinking and practice. Yet, many tools fall short of carefully exploring changes at the environmental and institutional level, and thus of shedding light on structural causes of gender inequality. Moreover, although many AR4D interventions focus on enhancing rural women's (and men's) resources—tangible and “countable” areas of change, such as income and assets, which are market-driven values (Narayan-Parker 2005; Cornwall 2014)—existing tools focus less on this dimension of empowerment. Finally, most tools explore instrumental agency (“power to”) rather than changes in intrinsic (“power within”) and collective agency (“power with”). This may be related to the difficulty of assessing the multiple dimensions of agency.

While all tools focus their assessments of agency mostly at the personal and/or relational level, the majority situate the analysis within the household, particularly looking at relations among spouses. Some tools rely on interviews of women only, whereas others rely on interviews with both women and men, often, but not exclusively, within the same household. Discussions of intersectionality in relation to measuring women's empowerment in AR4D remain, surprisingly, limited.

Quantitative tools for assessing women's empowerment in food systems use an etic (externally defined) perspective when defining or conceptualizing empowerment, with some exceptions. In contrast, the qualitative tools mostly, but not exclusively, use an emic perspective (the perspective of the respondent herself). Combining qualitative and quantitative methods in the measures themselves is less common, although recent metrics integrate qualitative data to a greater extent or use it for triangulation. For example, the pro-WEAI (Malapit et al., 2019a) has a suite of associated qualitative protocols, and Jayachandran et al. (2021) used qualitative methods and machine learning to recommend a smaller set of questions to measure empowerment in rural India.

## 4 Progress on women's empowerment and gender equality

### 4.1 Evolution of the conceptualization and measurement of women's empowerment

Tremendous progress has been made since 2010 on the measurement of women's empowerment in the context of agriculture-dependent LMICs, with great improvement, in particular, on the direct, quantitative measurement of different aspects and levels of women's agency (Desai et al., 2022; Elias et al., 2021). Direct approaches to measuring women's agency aim to measure the expression of agency, while indirect approaches aim to measure the material or economic resources that shape women's ability to exercise agency (Desai et al., 2022). The latter approaches have traditionally been viewed as being relatively straightforward to implement, as information on access to many resources is routinely collected in household surveys (e.g., land ownership, educational attainment, or employment). Different disciplines traditionally prioritized the measurement of different aspects of agency. For example, psychologists have tended to measure intrinsic agency through related concepts, such as self-determination and self-efficacy (Bandura 1997; Ryan and Deci 2000), whereas economists, rooted in intrahousehold bargaining theory, have focused on assessing instrumental agency by measuring women's participation in intrahousehold decision-making (Laszlo et al., 2020; Doss 2013). Interdisciplinary approaches have gained ground since 2011, including those using multidimensional indices of women's empowerment.

Launched in 2012, the WEAI measures women's agency and inclusion in agriculture across five domains—production, resources, income, leadership, and time—and is calculated based on interviews with women and men from the same households (Alkire et al., 2013). The WEAI comprises two subindices: 1) the Five Domains of Empowerment index (5DE), which measures women's empowerment at the individual level, and 2) the Gender Parity Index (GPI), which directly compares the empowerment of women and men from the same households. Used in 58 countries and by 243 organizations as of December 2022, WEAI data provide a comprehensive picture of women's empowerment in agriculture and the empowerment gap between men and women across continents and contexts.

Prior to the development of the WEAI, most quantitative metrics of women's empowerment had been unidimensional (i.e., focused on measuring a single aspect of agency) or indirect (i.e., focused on measuring women's access to material or economic resources) and were often calculated based on country-level statistics, rather than self-reported, individual-level data. No existing metric exclusively focused on measuring women's agency within the agricultural sector.

Following the launch of the WEAI, multidimensional empowerment indices based on the Alkire and Foster (2011) methodology—a counting method for measuring multidimensional poverty—using individual-level data have proliferated. Some are directly related to the WEAI and measured using similar survey questions and indicators. Abbreviated WEAI (A-WEAI) measures the same domains of empowerment as WEAI using a subset of the original indicators. Project-level WEAI (pro-WEAI) combines qualitative and quantitative data and shares a core set of common indicators with WEAI and A-WEAI but includes additional indicators to improve its ability to track empowerment impacts of agricultural interventions. More recently, pro-WEAI for health and nutrition (pro-WEAI + HN; Heckert et al. 2022) and pro-WEAI for market inclusion (pro-WEAI + MI) propose additional specialized indicators, which complement the set of standard indicators included in pro-WEAI and measure agency related to health and nutrition decisions and value chain activities, including the empowerment environment and factors such as sexual harassment in the workplace.

Other recent multidimensional empowerment indices use the same underlying Alkire-Foster methodology but focus on measuring empowerment in different domains and/or utilize different survey questions than the WEAI family of indices (see Fig. 1 for several examples). The Women's Empowerment in Fisheries Index (WEFI) adapts the WEAI to a fisheries-dominant context, in addition to including a gender-norms component (Cole et al., 2020). The Women's Empowerment in Livestock Index (WELI) adapts the WEAI to settings where livestock farming is the dominant form of livelihood and adds a domain on decisions related to nutrition (Galiè et al., 2019). The Women's Empowerment in Nutrition Index (WENI; Narayanan et al., 2019) and abbreviated WENI (A-WENI; Saha and Narayanan, 2022) use the Alkire-Foster methodology but are otherwise distinct from the WEAI. WENI and A-WENI measure women's empowerment in four domains, food, health, fertility, and institutions, utilizing different survey questions than the WEAI. Notably, unlike the WEAI family of indices, the WEFI, WELI and WENI do not collect data from men and thus do not provide direct estimates of the empowerment gap between men and women. Not collecting data on men in household surveys on empowerment is a missed opportunity both to assess the extent of the gender gap in empowerment and to identify whether disempowerment is due to gender or factors that affect the whole household (such as caste in South Asia). In impact evaluation applications, data on men is also helpful to be mindful of negative impacts on men's empowerment that might generate a backlash against women's empowerment efforts.

Others recent indices use publicly available data from Demographic and Health Surveys (DHS), including the Survey-Based Women's Empowerment Index (SWPER; Ewerling et al., 2017), SWPER Global (Ewerling et al., 2020) and Female Empowerment Index (Rettig et al., 2020). Another survey-based index to measure empowerment across three domains (choices, values, and norms) was recently developed and tested using data from India (Maiorano et al., 2021).

The aforementioned indices mostly use an etic (outsider) perspective when defining empowerment, though some used qualitative methods during the index development stage. In contrast, Oxfam GB's Women's Empowerment Index employs an emic (insider) perspective to curate a set of indicators, used to construct the index, that represent the characteristics of an “empowered woman” in the particular socio-economic context under analysis (Lombardini et al., 2017). Qualitative tools have also been developed for measuring empowerment from an emic perspective, including the GENNOVATE Ladder of Power and Freedom (Petesch et al., 2018) and CARE's Gender Indicator Monitoring Toolkit (Hillenbrand et al., 2015).

### 4.2 Data

The *World Development Report 2012* identified the availability of “gender-relevant data” as a key challenge for advancing gender equality, noting that “knowledge about what happens within households continues to be, at best, insufficient and, at worst, nonexistent” (World Bank 2011, 369). In recent years, several actions have been taken to close gender data gaps. The World Bank's Living Standards Measurement Studies—Integrated Surveys on Agriculture (LSMS-ISA) program expanded coverage to eight countries in sub-Saharan Africa. LSMS-ISA surveys are among the richest sources of timely and comprehensive data on agriculture—including information on women's control over assets and participation in decision-making on important agricultural decisions—in the region and have been instrumental in pushing forward new research on gender and agriculture (e.g., see Kilic et al., 2015). The LSMS Plus program was launched to enhance the availability and quality of intrahousehold survey data collected in LMICs on key dimensions of men's and women's economic opportunities and welfare (Kilic et al., 2021; Hasanbasri et al., 2021, 2022). Nevertheless, nationally representative data on women's empowerment continue to be scarce.

While there are global gender indices, such as the Global Gender Gap Index and SDG Gender Index, data on women's empowerment exist predominantly at the subnational level, thus there are no comparable indices for tracking changes over time in women's empowerment or comparing patterns across countries. The DHS program, which covers a wide range of countries, is a widely used source of data on decision-making but focuses more on the reproductive, rather than the productive, sphere. The development of a streamlined Women's Empowerment Metric for National Statistical Systems (WEMNS) for inclusion in national-level agricultural surveys as part of the 50x2030 Initiative is ongoing (https://www.50x2030.org/). The multilaterally funded 50x2030 Initiative aims to build capacity and improve the quality of national-level agricultural data collection in 50 countries by 2030. The wide-scale integration of WEMNS in these surveys would represent a major step forward in monitoring global progress on women's empowerment in agriculture and food systems and documenting progress toward SDG5.

## 5 What works to close the empowerment gap?

**Fig. 2:**
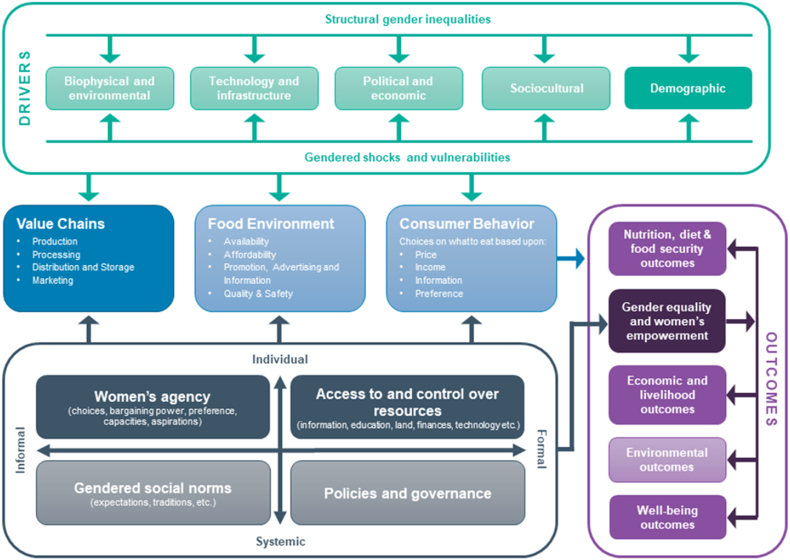
The gender and food systems framework.

Source: Myers et al. (2023), adapted from Njuki et al. (2022)

Understanding the factors that affect empowerment can help to design and implement appropriate interventions to close the empowerment gap between men and women. We draw on the conceptual framework from Njuki et al. (2022) (Fig. 2) to illustrate the relationships between various factors associated with empowerment and an evidence review described in Annex 1. We use these results to interpret the findings from impact evaluations of a portfolio of agricultural development projects with women's empowerment objectives and a systematic review of livestock interventions and empowerment.

### 5.1 Evidence from a portfolio of agricultural development projects

Impact evaluations of projects with explicit women's empowerment objectives are important sources of evidence on what works to empower women and close the empowerment gap. We draw on a synthesis of impact evaluations conducted across the portfolio of the Gender, Agriculture and Assets Project, Phase 2 (GAAP2) (Quisumbing et al., 2022). The GAAP2 portfolio comprises 13 agricultural development projects that co-developed the pro-WEAI (Malapit et al., 2019a) and used it to evaluate their projects' impacts on women's empowerment and gender equality. Pro-WEAI has three domains and 12 indicators. The instrumental agency (power to) domain has the most indicators, including: (1) productive decisions, (2) asset ownership (including land), (3) access to credit and financial services, (4) control over the use of income, (5) work balance and (6) visiting important locations. The intrinsic agency (power within) domain has four indicators: (1) autonomy in income decisions, (2) self-efficacy, (3) attitudes towards intimate partner violence against women and (4) respect within the household. Finally, the collective agency (power with) domain has two indicators: (1) group membership and (2) membership in influential groups.

All projects aimed to improve women's empowerment and nutrition outcomes, and some projects also aimed to improve incomes. Strategies used to empower women were broadly classified as: (1) providing goods and services, (2) strengthening organizations, (3) building knowledge and skills, and (4) influencing gender norms—though there was considerable variability in content of programming within each of these categories (Table 1). Outcome indicators used were the aggregate and individual indicators that comprise pro-WEAI (Malapit et al., 2019a, Malapit et al., 2019b).

**Table 1:** Activity areas and specific activities to empower women in GAAP2 projects.

Activity area	Specific activity	No. of projects using the activity as part of their strategy
Provide goods and services	Direct provision of goods/assets to beneficiaries	7
	Direct provision of services to beneficiaries	5
	Indirect provision by supporting availability, quality, or access	2
Strengthen organizations	Form/strengthen groups or other organizations (such as enterprises)	8
	Form/strengthen platforms or networks that link organizations	1
Build knowledge and skills	Agricultural training and extension	10
	Business and finance training	6
	Nutrition education	8
	Other training	4
Influence gender norms	Awareness raising about gender issues and their implications	3
	Community conversations to identify community solutions to gender issues	8

Adapted from: Johnson et al., (2018), p. 13.

Most GAAP2 projects provided goods and assets to beneficiaries (e.g., goats, financial services, improved seeds, technology packages) or facilitated the acquisition thereof (e.g., small-scale irrigation pumps). Although one expects this type of project strategy to affect instrumental agency indicators, such programs could potentially affect aspects of intrinsic agency. For example, Hillesland et al. (2022) found that a microfinance intervention delivered through rural savings and credit associations in Oromia, Ethiopia had a positive impact on the *respect among household members* indicator for beneficiaries who were able to maintain access to credit through the microfinance intervention between the baseline and endline.

Most projects also used group-based approaches. Membership in these groups can affect aspects of collective agency and provide access to different types of resources such as information, technology, credit, and other inputs. An impact evaluation of a nutrition-intensification platform, layered on an existing self-help group platform run by a large Indian nongovernmental organization in five states of rural India, found that self-help group membership has a significant positive impact on aggregate measures of women's empowerment and reduces the gap between men's and women's empowerment scores (Raghunathan et al., 2019; Kumar et al., 2021). In Burkina Faso, savings group members who received a comprehensive intervention package reported an increase in the average number of empowerment indicators in which they were adequate, while the comparison group saw a decrease in average adequacy over time (Crookston et al., 2021).

Training and building of knowledge and skills were also important parts of the GAAP2 projects’ strategies; evidence suggests that the mode of providing extension matters. For example, an impact evaluation of a pilot project in Bangladesh that randomized the provision of agricultural extension, nutrition behavior change communication, and gender-sensitization to husbands and wives jointly (Quisumbing et al., 2021a) found that all types of training, regardless of content, had positive empowerment impacts. Discussions with project staff suggest that these impacts arose because information was provided to husbands and wives when they were together, instead of separately as in previous programs.

Approaches to changing gender norms varied across the portfolio, including both one-way "awareness raising” and two-way community conversations to identify community solutions to gender issues (Johnson et al., 2018). Particular norms discussed included women's decision-making, time burdens, land ownership, mobility, market access, violence against women. Some projects worked only with women (such as the self-help group project in India), whereas two projects in Bangladesh worked with both women and men, as well as with community leaders and influential household members.

**Fig. 3:**
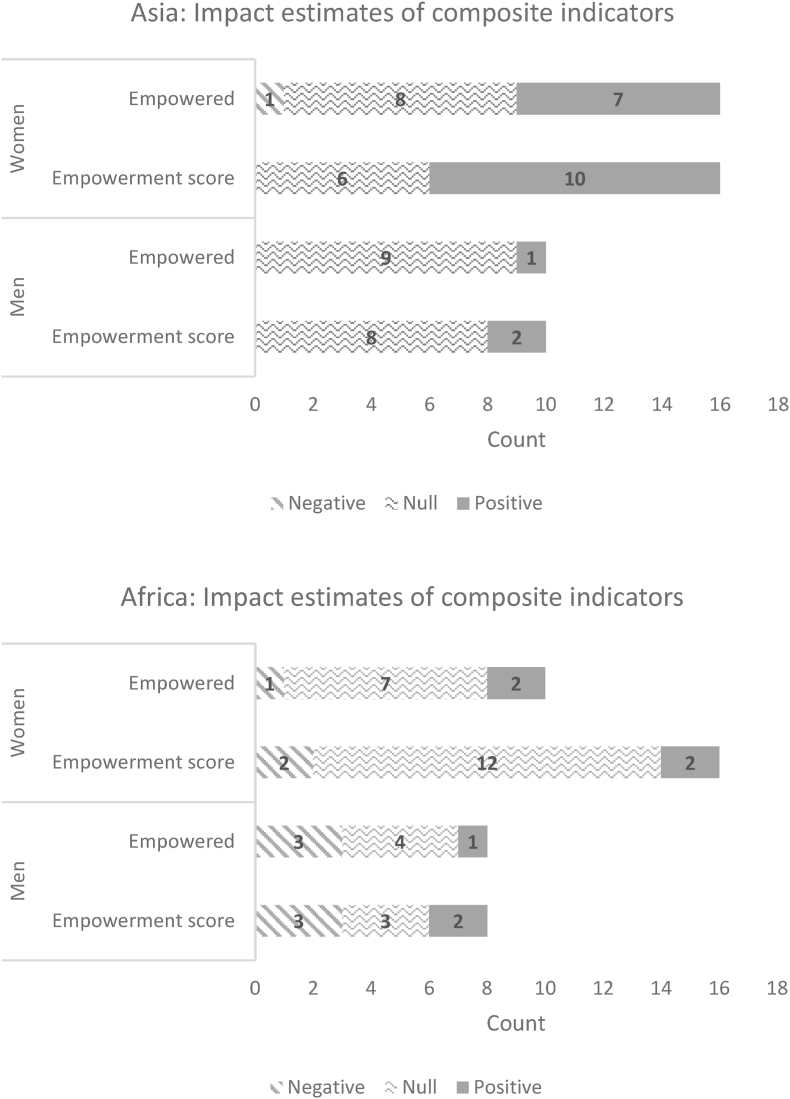
Distribution of impact estimates on whether empowered and empowerment score, GAAP2 portfolio Number of estimated coefficients: Asia: Women: 16; Men: 10; Africa: Women: 16; Men: 8. Count refers to the number of estimated impact coefficients across treatment arms in the GAAP2 portfolio (where measured). Definition of variables: Empowered denotes whether the individual is empowered (binary): An individual is defined as empowered if they achieved at least an empowerment score of 80% (A-WEAI) or 75% (pro-WEAI) Empowerment score (continuous): This is the proportion of indicators in which a respondent is adequate.

Source: Quisumbing et al. (2022).

**Fig. 4:**
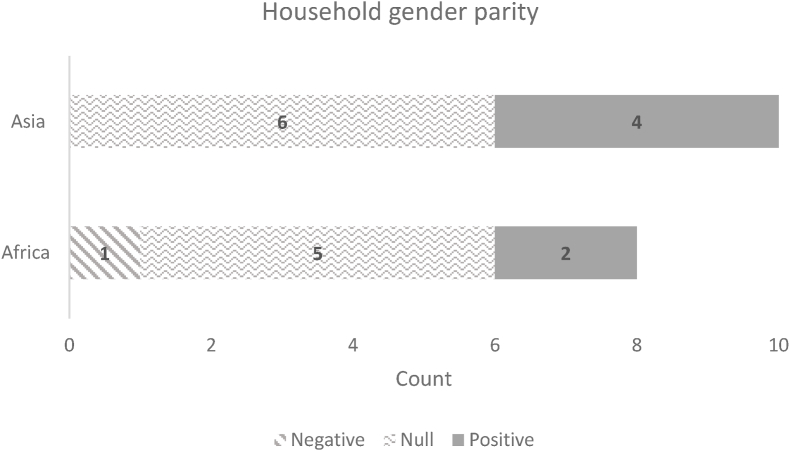
Distribution of impact estimates on whether the household achieved gender parity Number of impact estimates: Asia: 10; Africa 8 Notes: Count refers to the number of estimated impact coefficients across treatment arms in the GAAP2 portfolio (where measured). Definition of variables: whether the household achieved gender parity.

Source: Quisumbing et al. (2022).

Fig. 3, Fig. 4, derived from Quisumbing et al. (2022), present the results for women's and men's empowerment status (whether the individual was empowered), their respective empowerment scores, and whether the household achieved gender parity. Although all these projects had empowerment objectives, most of the coefficient estimates on the women's and men's empowerment indicators were statistically insignificant (Fig. 3). Moreover, most projects did not have a statistically significant impact on gender parity (Fig. 4).

Because the pro-WEAI comprises several indicators that may offset each other, changes in the aggregate index may mask changes in the component indicators. For example, an increase in the ability to make productive decisions may be counterbalanced by a deterioration in the work balance indicator (an increase in workload). The changes in the individual indicators offer more guidance to program designers and implementers because these can be directly affected by the program (e.g., the types of assets a woman owns).

**Fig. 5:**
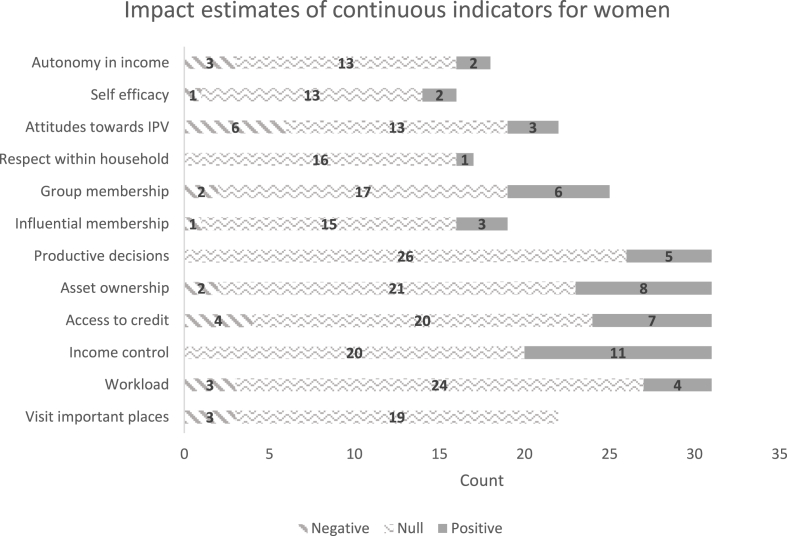
Distribution of estimated coefficients on women’s continuous indicators. Notes: Count refers to the number of estimated impact coefficients across treatment arms in the GAAP2 portfolio (where measured). Y axis lists the component indicators of pro-WEAI, continuous version.

Source: Quisumbing et al. (2022)

**Fig. 6:**
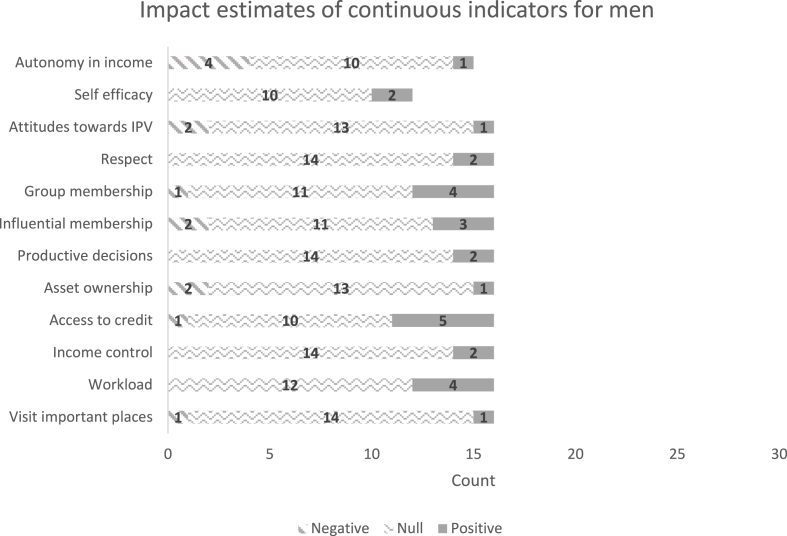
Distribution of estimated coefficients on men’s continuous indicators. Notes: Count refers to the number of estimated impact coefficients across treatment arms in the GAAP2 portfolio (where measured). Y axis lists the component indicators of pro-WEAI, continuous version.

Source: Quisumbing et al. (2022).

Fig. 5, Fig. 6 show the distribution of the estimated effect sizes on the continuous indicators, for women and men, respectively. These are standardized coefficients that divide the estimated coefficient by the standard deviation of the dependent variable. Most of the significant impacts are on instrumental agency indicators, possibly because these are more easily targeted and monitored by projects. Several instrumental agency indicators are significantly affected: (1) the types of activities for which the woman controls income, (2) the types of assets she controls (including land), and (3) the types of credit or financial services on which she decides. Reflecting on the group-based approaches used in these projects, there are positive impacts on the types of groups to which a woman belongs. Very few projects have statistically significant impacts on aspects of intrinsic agency, possibly because they may not have been directly targeted, or more realistically because intrinsic agency is linked to normative change, which may be slow and undetectable by quantitative indicators within the short time frame of an impact evaluation. Qualitative studies do find examples of increases in women's self-confidence and other aspects of intrinsic agency, but they are not necessarily the same across projects. Qualitative studies also show signs that gender norms may be changing, albeit slowly. Although there were very few significant impacts on men's indicators, since most of these projects were targeted to women, it is important to note any negative impacts on men, because they could signal potential backlash against women's empowerment projects. For example, men participating in an agricultural value chain project in Bangladesh experienced increased workload, like men in a savings and credit project in Burkina Faso and those who lost access to credit in Ethiopia. Regressions of the projects' impact coefficients on the presence of a specific type of strategy, controlling for region, indicate that projects with a capacity-building strategy were associated with larger estimated impacts on women's credit sources and the number of locations she can visit (Quisumbing et al., 2022). Surprisingly, projects with strategies to change gender norms did not have any significant impacts on instrumental, intrinsic, or collective-agency indicators. However, norm change is a long-standing process that may require months or years to yield a measurable difference in norms, and it may be unreasonable to expect significant impacts within the limited time frame of the impact assessments. Nevertheless, findings from seven qualitative studies of projects within the portfolio reveal that beneficiaries perceived capacity-building projects as having a strong, positive influence on their self-efficacy.

Although no strategies showed any statistically significant impact on the size of the impact estimates on the collective agency indicators, the null results could reflect lack of power owing to small sample sizes. Indeed, the qualitative findings indicate that many strategies were effective because they were delivered in a group-based format. These results highlight the challenges for projects that aim to contribute to women's empowerment. Deliberate strategies designed to contribute to women's empowerment (as opposed to just reaching or benefitting women) are important, but they need to be adapted to the context and implemented carefully. Further work is needed to identify what works, under what conditions and through what mechanisms. Consistent ways of measuring empowerment are an important first step toward building this knowledge base; qualitative research can help understand the context and mechanisms.

### 5.2 Livestock interventions and women's empowerment: what works

Women are the majority of small-scale livestock keepers in many LMICs and livestock play key roles in supporting livelihoods, nutrition, social status, and resilience (Randolph et al., 2007). Thus, livestock can provide key entry points to support women's empowerment. Women can own livestock—particularly smaller species—more easily than other assets (such as land and machinery); they can control the revenues generated from their livestock often without consulting men; livestock help women satisfy their traditional role as nutrition providers by providing animal source foods on a daily basis; women can invest in livestock to build their asset base in the absence of other financial institutions accessible to them; and finally, women can use their livestock to address crises by selling them in case of an urgent need for cash or keeping them in case of divorce (Galiè et al., 2022a). Livestock businesses, like the sale of milk and eggs, can also provide income-generating opportunities that are often scarce for rural or peri-urban women (Galiè et al., 2022b).

However, regressive gender dynamics and norms, if not addressed, reduce the empowerment potential of livestock. Men own larger and more valuable species than women do; women do not access animal health services, with negative impacts on the productivity of their animals (Enahoro et al., 2021); women tend to lose control over livestock-generated income in favor of men when this becomes lucrative (Tavenner et al., 2019). Market-oriented livestock farming requires business interactions with men outside their kinship networks, which women are discouraged from by long-standing tradition in contexts characterized by norms restricting women's mobility; this reduces their access to input and output services, markets, and other income-generating opportunities (Price et al., 2018; Galiè et al., 2022b).

A scoping review conducted in 2021 on the impact of livestock interventions on women's empowerment (and gender equality) identified 106 studies on the topic (Baltenweck et al., 2021). The authors adopted *decision-making*, *division of labor,* and *control over assets* as three broad outcomes to identify changes in women's empowerment. The most common livestock interventions that positively impacted women's empowerment included cooperatives and groups (e.g., supporting the formation of dairy cooperatives or brooder groups), followed by extension (e.g., provision of animal health or forage advice and inputs), training (e.g., on the benefits of artificial insemination or animal health practices), education, and productivity enhancing interventions (e.g. new feed varieties or breeds) (Table 2).

**Table 2:** Livestock interventions (number of interventions recorded in the 106 included studies).

Types of interventions	Number of studies
Groups/cooperatives	49
Extension, training, education	39
Productivity or husbandry	30
Access to output markets	27
Asset transfer	25
Access to inputs and services	25
Loans, microcredit	14
Total number of studies	106

**Fig. 7:**
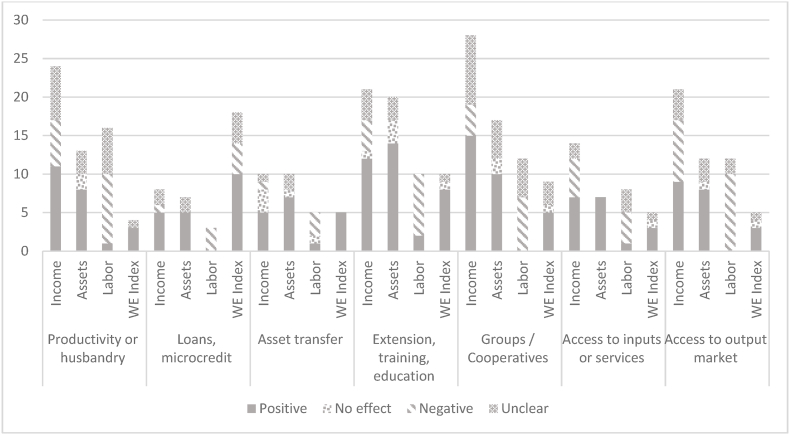
Impact of livestock interventions on access to and control over income, access to and control over assets, and workload.

Source: Based on Baltenweck et al. (2021).

Comparing the impacts of each type of intervention across all the *domains* of empowerment, the review found that loans and microcredit had the most positive impact across all measured indicators of empowerment, followed by asset transfer and extension, training and education (Fig. 7). Loans/microcredit, asset transfer/extension, and training/education had the highest impact on both *access to and control over income from livestock* and *access to and control over livestock assets*, and a negative impact on *women's labor and workload*. Most interventions generally had negative impacts on *women's labor and workloads*. When comparing the impacts of each type of intervention on each *indicator*, extension, training, education, and groups/cooperatives were the interventions that most positively affected both *access to and control over income from livestock* and *access to and control over livestock assets*, while access to output markets emerged as the least positively impactful intervention overall.

Of note is that half the studies report cases that bundle interventions. For example, there are seven studies of asset transfer and extension. Caution should therefore be made when interpreting these ‘individual intervention’ effects. When analyzing these ‘packages’ of interventions, ‘productivity and extension’ studies usually report a positive effect on women's empowerment.

Provision of animal extension services emerged as a key intervention supporting women's empowerment. This is consistent with the evidence that women generally have limited access to such services that are necessary to have healthy and productive animals (Dolapo et al., 2021); and that supporting women's access to animal services is likely to enhance their empowerment (Omondi et al., 2022). Training and education also appear to be effective interventions to support women's empowerment. Together with boosting women's knowledge of animal husbandry, formal education may help make women's role as livestock keepers and their associated knowledge visible in households and communities, with positive consequences for their decision-making and access to services (Galiè et al., 2012).

The positive impacts of group membership and cooperatives in terms of social capital and women's empowerment have been shown in the literature (see e.g. Ferguson and Kepe, 2011). The negative impacts of all interventions on women's labor speak to the fact that, unless gender norms associating women with household chores are addressed, women will continue to bear the time burden of livestock interventions, particularly those that promote intensification of production (which in turn requires additional labor inputs). Such time burdens are most likely unpaid and invisible, particularly in gender-blind studies.

Jumba et al. (2020) show how gender norms and dynamics influencing the distribution of labor and control over income may interact with a livestock vaccine intervention. In the studied communities of Tanzania, women provided labor for the livestock and men marketed the livestock and controlled the income earned. Because the livestock vaccine reduced cattle mortality, women's workload increased as they had to provide for the larger herds. This increased workload, however, did not benefit women because only men sold the cattle and controlled the income. This decline in mortality rates due to greater access to livestock vaccine not only disempowered women, but also reduced their support for the vaccine, and consequently, its adoption by households. In contrast, Galiè and Kantor (2016) showed how, in some pastoral communities of Tanzania, women welcomed an increase in their labor associated with the introduction of improved goat breeds. These breeds had to be kept in the courtyard, given their susceptibility to disease. The courtyard was a space controlled by women, because the men spent most of their time in the savanna migrating with the herd of local breeds. As a result, women not only looked after the new breeds but also controlled the increased milk production, which they used to feed the children, and earned some cash from selling milk. Clearly, local context affects the way gender dynamics and norms interact with livestock interventions and affect women's empowerment.

Finally, given the evidence that livestock development which successfully supports women's empowerment hinges upon changes in both technical (e.g., better breeds) as well as social institutions (e.g., more equitable gender norms), the impact of packages of interventions needs to be better studied.

## 6 Additional benefits to closing the empowerment gap

Although most development actors acknowledge the intrinsic value of women's empowerment and gender equality, evidence of additional social benefits helps justify increased attention to these goals.

A recent review of studies using WEAI metrics (empowerment score, intrahousehold empowerment gap, component indicators), provides evidence on how gender equality and women's empowerment influence food systems outcomes (Myers et al., 2023). Of 30 peer-reviewed papers that analyze WEAI as an explanatory variable, almost three-quarters focus on relationships between empowerment and nutrition, individual diets, household food security, and WASH; ten analyze economics and livelihoods outcomes (agricultural production); and two focus on well-being outcomes. Two studies (Clement et al., 2019 in Nepal; Wouterse, 2017 in Niger) overlap economic and food security outcomes, and one study in Bangladesh (Malapit et al., 2019a, Malapit et al., 2019b) covers both nutrition and well-being. Six studies tackle multiple themes of nutrition, diets, food security, and WASH. However, no existing study analyzed environmental and natural resource outcomes, except for adoption of soil management, which is an important gap given women's important role in natural resource management (Meinzen-Dick et al., 2014). Although (James et al., 2021) find some evidence of a positive relationship between women's engagement of women and environmental outcomes, explicit attention to women's empowerment has been lacking in these studies.

Table 3 summarizes the consistency of the evidence around women's empowerment and its associations with food systems outcomes based on the number of studies and their degree of agreement. Although all studies included in our synthetic review included covariates, most of these are associational; thus estimated effects should not be interpreted as causal. Overall, the greatest amount of evidence and strongest agreement is that women's empowerment is associated with improved *diets* and *child nutrition*. There are also strong, positive relationships between women's empowerment and *life satisfaction*, *educational outcomes*, and *WASH*, though there is relatively less evidence demonstrating these associations. Interestingly, the relationship between *women's nutrition* and women's empowerment is not as well researched as *child nutrition*, and the mixed results on *women's nutrition* illustrate the potential trade-offs that women may face in their multiple roles as income earners and caretakers of their households' (and their own) food security and nutrition.

**Table 3:** Women's empowerment in relation to development outcomes.

		Amount of evidence		
		Low (1–3 studies)	Medium (4–6 studies)	High (7–9 studies)
Degree of agreement	Low			
	Medium		Women's nutrition	Household-level food security Agricultural production
	High	Life satisfaction Educational outcomes WASH		Diets Child nutrition

Note: This review is based on papers that use WEAI or its variations.

### 6.1 Children's diets and nutrition

Our most consistent finding is that women's empowerment, whether measured using the empowerment score or the WEAI component indicators, is significantly positively associated with many children's dietary and nutrition outcomes (Bonis-Profumo et al., 2021; Cunningham et al., 2015, 2019; Holland and Rammohan 2019; Malapit and Quisumbing 2015; Quisumbing et al., 2021b; Zereyesus 2017). While analyses using the aggregate empowerment score generally show positive associations, disaggregating empowerment into the component indicators that capture different aspects of agency shows that different indicators matter in different contexts. For example, in Bangladesh, Holland and Rammohan (2019) found that *input in productive decisions* and *speaking in public* are positively associated with children's height-for-age z-scores (HAZ) and with lower probability of stunting. In Nepal, Cunningham et al. (2015) found that *satisfaction with leisure time*, *access to and decisions regarding credit* and *autonomy in production* were positively associated with length-for-age z-scores (LAZ) for children under 2, while for children under 5, Malapit et al. (2015) found that control over income is positively associated with HAZ. In Timor-Leste, *group membership* and *asset ownership* are positively associated with children's dietary diversity (Bonis-Profumo et al., 2021), whereas greater *workload* is associated with higher children's dietary diversity in Bangladesh, Cambodia, Ghana, Mozambique, Nepal and Tanzania (Quisumbing et al., 2021b). These results suggest that different types of intra-household decisions (e.g., production, credit, assets) may be relevant for directing resources towards children's diets and nutrition. Access to social support networks, community activities or groups may also facilitate knowledge and skills transfer, which, coupled with self-esteem and autonomy, may enable women to act on information received and create an enabling environment for child nutrition and growth (Cunningham et al., 2019).

In addition to women's empowerment, intrahousehold gender equality matters for children's dietary and nutrition outcomes. Several studies have found that greater equality within the household, measured by a reduction in the male–female intrahousehold empowerment gap, is positively correlated with HAZ in Nepal (Malapit et al., 2015), Ghana (Malapit and Quisumbing 2015), and a six-country study including Bangladesh, Cambodia, Ghana, Nepal, Mozambique, and Tanzania (Quisumbing et al., 2021b). Greater intrahousehold equality is also positively associated with child dietary diversity in Nepal (Malapit et al., 2015) and exclusive breastfeeding in Bangladesh, Cambodia, Ghana, Mozambique, Nepal, and Tanzania (Quisumbing et al., 2021b). Another study by Malapit et al. (2019b) in Bangladesh analyzes gender gaps not only in the overall empowerment scores between men and women within the same household, but also the male–female differences in the component indicators. They find that these empowerment gaps are weakly correlated with children's nutrition outcomes, but with differences across boys and girls. For example, an increase in women's credit decision-making (smaller gender gap), is positively associated with girls' HAZ, while an increase in women's participation in groups (smaller gender gap), is positively associated with WAZ, favoring boys rather than girls. Thus, it does not always follow that women's empowerment benefits girls; in societies where there is son preference, more empowered women may differentially invest in boys. In Bangladesh, using the same data set, Sraboni and Quisumbing (2018) also found a positive association between women's empowerment and diet quality of individuals within the household, but with varying strength across the life course. Women's empowerment is positively and significantly associated with adult men's and women's dietary diversity and nutrient intakes but does not benefit all individuals within the household equally, with gender bias favoring boys emerging in adolescence.

### 6.2 Women's diets and nutrition

Despite the benefits to children's diets and nutrition associated with women's empowerment and intrahousehold gender equality, tradeoffs with women's own diets and nutrition may exist. Several studies document significant associations between women's empowerment indicators and women's dietary diversity scores (Bonis-Profumo et al., 2021; Malapit et al., 2015; Onah et al., 2021; Wouterse 2017). However, the component indicators show mixed results.

For example, in Ghana, Ross et al. (2015) did not find a significant relationship between women's aggregate empowerment score and women's health status, as measured by body mass index (BMI) and dietary diversity score (DDS). However, when the empowerment score was disaggregated into its component indicators, they found that five indicators were significantly associated with better health status for women but with offsetting signs. *Asset ownership*, *credit decisions*, *group membership* and *satisfaction with leisure* were all positively associated with women's health status, but *autonomy in production* had an unexpected negative relationship. Upon further investigation, Ross et al. (2015) found that women in higher income groups had significantly less *autonomy in production*. As women increase their economic activities and contribute more income to the household, they may feel pressure to make production decisions based on others' expectations to avoid conflict.

Similarly, the most striking result from the six-country study by Quisumbing et al. (2021b) in Bangladesh, Cambodia, Ghana, Nepal, Mozambique, and Tanzania was the lack of significant association between the aggregate empowerment measures and most of the women's nutritional outcomes. However, analysis of the component indicators revealed significant associations with offsetting signs, suggesting potential trade-offs between different domains of empowerment. Specifically, they find that *speaking in public* was associated with improved women's dietary diversity, but the *number of types of agricultural decisions*, *autonomy in production*, *number of types of agricultural assets owned* and *number of types of income decisions* were all associated with less diverse diets for women. *Speaking in public* may be capturing social capital and self-esteem (Holland and Rammohan 2019) that may be more directly linked to women's own consumption compared with the other indicators. On the other hand, greater intrahousehold equality (smaller gender gap), a greater *number of types of agricultural decisions*, more *autonomy in production* and a higher *workload* were all associated with lower BMI, while comfort with *speaking in public* and *satisfaction with leisure* were associated with higher BMI. These tradeoffs may arise because women's increased participation in agriculture, which improves some components of the women's empowerment score, may also increase workload, which may reduce BMI in low-BMI populations (Quisumbing et al., 2021b).

### 6.3 Household food security

Women's empowerment also appears to be positively correlated with household food security, measured by the household dietary diversity score (HDDS). Several studies have found positive associations between HDDS and women's aggregate empowerment scores in Bangladesh (Sraboni et al., 2014; Holland and Rammohan 2019) and Niger (Wouterse 2017). Consistent with the findings on diets and nutrition outcomes, different component indicators matter in different contexts (Chitja and Murugani 2018; Clement et al., 2019; Quisumbing et al., 2021b; Seymour et al., 2019). Intrahousehold gender inequality also matters, according to one study in Bangladesh, which found that larger intrahousehold empowerment gaps are associated with marginally lower HDDS among nonpoor, time-poor, and doubly-poor (both income- and time-poor) households (Seymour et al., 2019). They estimate that attaining full gender equality can improve household dietary diversity by 0.5 food groups, which may be more meaningful for the doubly-poor who consume on average 1.5 fewer food groups than nonpoor and time-poor households (Seymour et al., 2019).

One study in Nepal that examined the share of vegetable and cereal production retained for home consumption (Clement et al., 2019) found that women who are adequate in *access to and decisions about credit* keep a significantly larger share of both vegetable and cereal production for home consumption. However, women who are adequate in *control over income* keep a significantly smaller share of vegetable production for home consumption. In this context, cereal production and sales are considered to belong in the man's domain, whereas homestead vegetable production and sales are in the woman's. Thus, women with greater control over income are more likely to sell more vegetables, given that homestead vegetable production and sales are an important—often only—source of rural women's incomes (Clement et al., 2019).

Overall, our review finds that increasing women's empowerment and closing empowerment gaps contribute to household food security, but household wealth, gender norms, and country-specific institutions are also of critical importance. Quisumbing et al. (2021b) found that household wealth and country characteristics account for the largest proportion of the variance in household and women's dietary diversity compared to the small share contributed by women's empowerment. This suggests that diets, nutrition, and food security outcomes cannot be expected to improve automatically when women are empowered without also addressing the underlying determinants of poor nutrition (Quisumbing et al., 2021b).

### 6.4 Agriculture

Several studies have found positive associations between various empowerment measures and agricultural production indicators (Anik and Rahman 2021; De Pinto et al., 2020; Diiro et al., 2018; Seymour 2017; Wouterse 2017, 2019). For example, in Niger, empowerment scores are positively associated with agricultural output (Wouterse 2017, 2019). Wouterse (2019) estimated that an increase of 1.0% in average empowerment would increase output by almost 1.0%. She also found that empowerment interacts positively with the value of agricultural equipment owned by the household and negatively with the use of fertilizer by the household (Wouterse 2019), and that empowered households are more likely to have zai pits, a climate change–adaptive land-preparation method also referred to as ‘planting pits’ (Wouterse 2017). Women's overall empowerment is also positively associated with production efficiency in Bangladesh (Anik and Rahman 2021) and among maize farmers in Kenya (Diiro et al., 2018). In Bangladesh, De Pinto et al. (2020) found that as women's *input in productive decisions* increased, less land was allocated to cereals and more to vegetables and fruits. Women's *participation in economic or social groups* is also positively associated with greater crop diversification, as measured by an increase in land allocated to vegetables and fruits and a decrease in land allocated to cereals (De Pinto et al., 2020).

Greater intrahousehold equality is positively correlated with production efficiency in Bangladesh (Anik and Rahman 2021; Seymour 2017). Seymour (2017) found that this result extended to plots jointly managed by women and their spouses, as well as to those that women do not actively manage.

Only two studies found potential trade-offs between empowerment and agricultural outcomes. Clement et al. (2019) found that in Nepal, women's *access to and decisions about credit* are both significantly correlated with lower wheat productivity and a greater share of cereals kept for own consumption. In Malawi, Mponela et al. (2021) collected 3 WEAI domain indicators (decisions about agricultural production; control over use of income; and access to and decision-making power about productive resources) and found that a 1.0 percentage point increase in an aggregate of these three domain indicators leads to a 0.33 percentage point increase in the area allocated to legumes but reduces the amount of organic manure applied, with higher elasticity of 2 percentage points. In both cases, the type of crop matters: cereals are generally considered men's crops in Nepal (Clement et al., 2019), while legumes are considered women's crops in Malawi (Mponela et al., 2021).

### 6.5 Other well-being outcomes

Two studies in Bangladesh investigated the relationship between women's empowerment and other well-being outcomes, such as life satisfaction and children's schooling. Hossain et al. (2019) found that life satisfaction among women and men is positively associated with aggregate empowerment as well as seven component indicators: *input in productive decisions*; *purchase*, *sale or transfer of assets*; *ownership of assets*; *access to and decisions about credit*; *control over use of income*; *leisure*; and *group membership*. The findings on child schooling are more nuanced. Malapit et al. (2019b) found that fathers' empowerment is positively associated with younger children's schooling, while mothers' empowerment is more important for girls' education and for keeping older boys and girls in school.

### 6.6 Summary

Overall, women's empowerment and gender equality, as measured by the WEAI indicators, is significantly associated with various development outcomes, but which aspects of empowerment are most important for improved outcomes vary across contexts. While some outcomes, such as children's diets and nutrition, are consistently positively associated with women's empowerment, important tradeoffs emerge. Increased engagement in agriculture may improve some aspects of empowerment, but also increase women's workloads on top of existing care and domestic work responsibilities.

We also uncovered some notable gaps. One is the absence of any studies on environmental and natural resource outcomes—a critical area that should be addressed by future research. Numerous conservation organizations and multilateral or donor organizations seek to increase women's participation in natural resource management because women have different knowledge and interests in the environment, but social norms often preclude women from decision-making, even about resource issues that affect them.

Second is the lack of evidence on other economic and livelihood outcomes beyond agricultural production, such as poverty status, income, employment, and wages. Although unsurprising given the WEAI's original focus on smallholder production, the development of the pro-WEAI for market inclusion (pro-WEAI + MI) tool expands coverage to include value chain activities beyond production. Third, very few studies cover other outcomes such as WASH, life satisfaction, children's schooling outcomes, and even women's own aspirations. Fourth, very few studies examine empowerment over the life cycle. Finally, because most of the 30 studies reviewed in this section are observational studies, we need more impact assessments to establish causality and to unpack the specific mechanisms through which empowerment leads to these changes. The latter point is particularly true for understanding the relationships between empowerment and agricultural outcomes, given the range of results observed in the literature and complexity of women's involvement in agricultural production.

## 7 Conclusions and policy recommendations

Our review of the evolution of women's empowerment and gender equality metrics shows that our understanding and conceptualization of women's empowerment affect what and how we measure. Current approaches to measuring empowerment have gone beyond exclusively emic (insider) and etic (outsider) views to those that draw on the strength of combined qualitative and quantitative approaches. However, most measures of women's empowerment are at the individual level and need to consider the household and community levels. Having a standardized measure of women's empowerment (like the WEAI) facilitates comparisons across geographies but needs to be contextualized and grounded using qualitative work. Standardized measures also facilitate comparison across projects to assess what approaches work to empower women and achieve gender equality, while qualitative work can help address *how* they work. Because factors correlated with women's empowerment are likely to vary by culture and context, interventions that aim to empower women and improve gender equality need adaptation to be effective.

While women's empowerment and gender equality have intrinsic value, the pursuit of these goals is most often justified because of their social benefits—better health, diet, and nutrition outcomes—and increased efficiency and agricultural productivity. Having better measures of women's empowerment also contributes to better and more rigorous analysis of the relationship between women's empowerment and gender equality and other development outcomes. Finally, measuring women's empowerment is not enough. Data on men's empowerment indicators are needed to track gender equality, to create awareness of any backlash against programs that aim to empower women, and to examine how reducing the empowerment gap contributes to development outcomes.

Our review of programs and projects suggests several recommendations for policy- and decision-makers designing and implementing gender-transformative policies and programs. First, intentionality is important: programs that seek to empower women should have deliberate strategies to contribute to their empowerment that are appropriate for their culture and context. Examining baseline data on the major sources of disempowerment, as well as the experiences of other projects in that region, can help design more empowering projects. Second, while group-based approaches have been effective in empowering women, we must recognize the risk of excluding the most vulnerable from group-based programs, the role of intra-group dynamics, and possible increases in work burden owing to the time required to participate in group activities.

Gender norms will not change by working with women alone. For programs to be gender-transformative, they must also involve men and change institutional structures. In some cultures, involving key decision-makers in the household and community (in-laws, traditional leaders) may be key to program success. Women-targeted programs should also be aware of potential trade-offs between women's involvement and time burden. Many well-intentioned programs unwittingly increase women's workload, with negative consequences for women themselves. The time vs. income tradeoff is important, and women and men may have different preferences over those resources given other responsibilities, such as caregiving.

Finally, efforts to measure different aspects of agency that might be affected by development interventions in market inclusion, health and nutrition, and livestock and to improve data collection at the individual level must continue. The new generation of empowerment metrics (WENI, WELI, WEFI) and specialized add-on modules for pro-WEAI (pro-WEAI + HN, pro-WEAI + MI) are examples of the former. Initiatives targeting the latter include closer attention to collecting data on men--to better track men's outcomes, gender gaps in empowerment, and potentially, changes in gender norms--and to scaling up the collection of empowerment metrics in nationally representative surveys and national statistical systems. Across these efforts, data collection should also include other variables that will enable the analysis of overlapping aspects of disadvantage (e.g., age, race, ethnicity) that intersect with gender.

## Declaration of competing interest

The authors declare that they have no conflict of interest.

## Figures and Tables

**Annex Table 1:** Number of included studies, study region, and species

	Cattle	Cattle and small ruminants	Small ruminants	Small ruminants and poultry	Poultry	Pigs	Multiple species	Total
America (Central and South)							2	2
Eastern Africa	24	3	2		1	1	8	39
Africa (Central, South, West)	2		1	1	4	2	2	12
South Asia	30	1	4		5		7	47
Asia (East, Southeast, West)	1		1			2	2	6
Total	57	4	8	1	10	5	21	106

Source: Baltenweck et al. (2021).

## Data Availability

No data was used for the research described in the article.

## Annex 1: Search and literature review strategy

### 1. Correlates of empowerment and relationships between empowerment and other outcomes

While the development of tools and approaches for measuring women's empowerment is an active research area (see Elias et al., 2021 for a review), this search focuses on the studies using the Women's Empowerment in Agriculture Index (WEAI) to permit meaningful comparisons of findings across studies. Recent reviews highlight the difficulty in making comparisons across studies owing to the wide variation in the approaches and operationalization of empowerment indicators (Santoso et al., 2019).

Between 2012 and 2022, the use of the WEAI has grown significantly to 243 organizations in 58 countries, providing a sizable set of studies for the review.

#### 1.1 Search methodology and inclusion/exclusion criteria

We conducted an initial search for all publications that cited the original WEAI paper, Alkire et al. (2013), from 2013 through June 2020 via Google Scholar. Out of the 626 total records found, we then restricted these publications to include only peer-reviewed literature published in English; working papers, theses, and reports were excluded. To facilitate the comparison of findings, we further restricted the papers included to only those that calculated a WEAI metric such as the 5DE, 3DE, GPI, a singular WEAI indicator, a subset of indicators, or a combination thereof. We did not include papers that used individual WEAI survey items or those that greatly adapted WEAI survey items and/or indicators. Nor did we include papers that did not report significance levels (p-values) of their quantitative findings. To account for papers published after June 2020, we conducted an additional search for publications that also cited the original WEAI paper or the original pro-WEAI paper, Malapit et al. (2019b), from 2020 through April 2022 via Google Search.

This search yielded 434 results, which we further restricted to only peer-reviewed literature in English and, as before, those that used a WEAI metric either from the original WEAI tool, A-WEAI or pro-WEAI. We further restricted these to journal articles and eliminated one in the post-screening stage because of incorrect econometric methodology. We added one more article on poverty transitions that we missed in our initial search. Summing both searches together, our literature review includes 30 papers that meet all criteria. Majority of the papers reviewed use observational data, and therefore the results reported should be interpreted as associational rather than causal. Moreover, many of the studies may not have implemented multiple hypothesis testing corrections. See section 6 for a discussion of related literature that provides evidence on the impacts of livestock interventions (Baltenweck et al. forthcoming) and a synthesis of impact evaluations conducted across the portfolio of 13 agricultural development projects under the Gender, Agriculture, and Assets Project, Phase 2 (GAAP2) (Quisumbing et al., 2022).

#### 1.2 Results

In section 6 we report on the 30 studies that examine how gender equality and women's empowerment, measured using WEAI metrics, influence other outcomes of interest— diet, nutrition and food security outcomes (including WASH), economic and livelihood outcomes (primarily in agriculture), and other well-being outcomes such as life satisfaction and children's education. None of the studies analyzed environmental and natural resource outcomes. See Myers et al. (2023) for complete results. As other empowerment metrics are developed, we expect to see more studies relating these measures with other development outcomes.

### 2. Scoping review of livestock interventions

This section draws on a scoping review conducted in 2021 and 2022 on the impact of livestock interventions on women's empowerment (and gender equality) that identified 106 studies on the topic, mostly published after 2010 (Baltenweck et al. forthcoming). Annex Table 1 provides an overview of geographical location and species characterizing these interventions.
